# Age-Related Decrease in Heat Shock 70-kDa Protein 8 in Cerebrospinal Fluid Is Associated with Increased Oxidative Stress

**DOI:** 10.3389/fnagi.2016.00178

**Published:** 2016-07-26

**Authors:** David A. Loeffler, Andrea C. Klaver, Mary P. Coffey, Jan O. Aasly, Peter A. LeWitt

**Affiliations:** ^1^Departments of Neurology, Beaumont Hospital-Royal Oak, Beaumont Health, Royal OakMI, USA; ^2^Departments of Biostatistics, Beaumont Hospital-Royal Oak, Beaumont Health, Royal OakMI, USA; ^3^Department of Neurology, St. Olav’s HospitalTrondheim, Norway; ^4^Department of Neurology, Henry Ford West Bloomfield Hospital, West Bloomfield TownshipMI, USA; ^5^Department of Neurology, Wayne State University School of Medicine, DetroitMI, USA

**Keywords:** aging, cerebrospinal fluid, HSPA8, 8-hydroxydeoxyguanosine, 8-isoprostane, oxidative stress

## Abstract

Age-associated declines in protein homeostasis mechanisms (“proteostasis”) are thought to contribute to age-related neurodegenerative disorders. The increased oxidative stress which occurs with aging can activate a key proteostatic process, chaperone-mediated autophagy. This study investigated age-related alteration in cerebrospinal fluid (CSF) concentrations of heat shock 70-kDa protein 8 (HSPA8), a molecular chaperone involved in proteostatic mechanisms including chaperone-mediated autophagy, and its associations with indicators of oxidative stress (8-hydroxy-2′-deoxyguanosine [8-OHdG] and 8-isoprostane) and total anti-oxidant capacity. We examined correlations between age, HSPA8, 8-OHdG, 8-isoprostane, and total antioxidant capacity (TAC) in CSF samples from 34 healthy subjects ranging from 20 to 75 years of age. Age was negatively associated with HSPA8 (ρ = –0.47; *p* = 0.005). An age-related increase in oxidative stress was indicated by a positive association between age and 8-OHdG (ρ = 0.61; *p* = 0.0001). HSPA8 was moderately negatively associated with 8-OHdG (ρ = –0.58; *p* = 0.0004). Age and HSPA8 were weakly associated with 8-isoprostane and TAC (range of ρ values: –0.15 to 0.16). Our findings in this exploratory study suggest that during healthy aging, CSF HSPA8 may decrease, perhaps due in part to an increase in oxidative stress. Our results also suggest that 8-OHdG may be more sensitive than 8-isoprostane for measuring oxidative stress in CSF. Further studies are indicated to determine if our findings can be replicated with a larger cohort, and if the age-related decrease in HSPA8 in CSF is reflected by a similar change in the brain.

## Introduction

Aging is the most consistent known risk factor for developing progressive neurodegenerative disease (Jeppesen et al., 2011). An important contributor to this risk is likely to be the increased oxidative stress that develops in the aging brain (Floyd and Hensley, 2002). Age-associated declines in protein homeostasis (“proteostasis”) mechanisms may also be involved. Oxidative mechanisms are likely to play a role in the formation of misfolded protein aggregates that are pathological hallmarks of age-associated neurodegenerative disorders, because they promote protein aggregation (Squier, 2001). Accumulation of misfolded and unfolded proteins in the endoplasmic reticulum (ER) causes “ER stress” which activates the unfolded protein response (UPR). If this response fails to reduce the protein load in the ER then cell death may result (Cuanalo-Contreras et al., 2013). A second proteostatic mechanism in addition to the UPR is autophagy, which removes damaged proteins and organelles by degrading them within lysosomes (Cuanalo-Contreras et al., 2013). Three types of autophagic processes are recognized (reviewed by Filomeni et al., 2015), namely macroautophagy (sequestration of damaged organelles into autophagosomes), microautophagy (engulfment of cytosolic material by lysosomes), and chaperone mediated autophagy (CMA), through which selected cytosolic proteins translocate to the lysosome for degradation without the requirement for vesicle formation (Cuervo, 2010). Autophagy can be organelle-specific, including mitophagy, reticulophagy, and ribophagy (Osellame and Duchen, 2014). Oxidative stress can activate autophagic processes including CMA (Kiffin et al., 2004).

Heat shock 70-kDa protein 8 (HSPA8, also known as Hsc70 and Hsc73) is a cytosolic molecular chaperone involved in multiple proteostasis mechanisms including CMA (Liao and Tang, 2014). We recently measured HSPA8 in cerebrospinal fluid (CSF) samples from patients with sporadic Parkinson’s disease (PD), PD patients carrying leucine-rich repeat kinase 2 (*LRRK2)* gene mutations, healthy control subjects carrying *LRRK2* mutations, and healthy controls without *LRRK2* mutations (Loeffler et al., 2016). *LRRK2* gene mutations are the most common cause of inherited PD (Vilas et al., 2016). We included, in that study, data from 23 healthy control subjects without *LRRK2* mutations ranging from 45 to 75 years old. At the same time, we measured HSPA8 in 11 younger control subjects ranging from 20 to 43 years of age who were not included in the study. Our unpublished results from these control subjects suggested associations between age and HSPA8 concentrations. The age-related changes in HSPA8 in the human CNS are unknown, and the literature contains conflicting reports with regard to its alterations with age in experimental animals (Unno et al., 2000; Calabrese et al., 2004; Gleixner et al., 2014). In this study, we present our findings with regard to age-related changes in CSF HSPA8, and the associations between this protein and oxidative stress biomarkers 8-hydroxy-2′-deoxyguanosine (8-OHdG) (Valavanidis et al., 2009) and 8-isoprostane (8-ISO) (Morrow et al., 1992) as well as total antioxidant capacity (TAC) (Bartosz, 2003).

## Materials and Methods

### Study Subjects

Lumbar CSF samples were obtained from 34 healthy subjects by Jan Aasly, M.D., Ph.D. (St. Olav’s Hospital, Trondheim, Norway), using the Parkinson’s Progression Markers Initiative (PPMI) biospecimen collection procedures (Parkinson’s Progression Markers Initiative, 2014). CSF was collected from fasting subjects between 8 and 9 AM; within 15 min of collection, it was centrifuged at 2000 × *g* for 10 min at room temperature, then frozen within 60 min of collection and stored at –80°C. The study was approved by the Regional Committee for Medical Research Ethics, Central Norway, for the procedures performed at St. Olav’s Hospital (subject recruiting and obtaining of CSF samples). Written informed consent was obtained from all subjects, in accordance with the Declaration of Helsinki, before lumbar punctures. The subjects were tested for and lacked known PD-related mutations in the *LRRK2*, *PARK2*, *PARK7*, *PINK1*, and *SNCA* genes, and had no detectable cognitive or other neurological impairments. The study was given exempt status by the Institutional Review Board of Beaumont Health (Beaumont Hospital-Royal Oak, Royal Oak, MI, USA) where measurements of HSPA8, 8-OHdG, 8-ISO, and TAC were performed.

### Measurements of HSPA8, 8-OHdG, 8-ISO, and TAC in CSF Samples

HSPA8 was measured with MyBioSource’s Heat Shock 70kDa Protein 8 (HSPA8) ELISA Kit (cat. # MBS2023476). The detection limit for this assay was stated by the manufacturer to be 0.128 ng/mL. 8-OHdG, 8-ISO, and TAC were measured with kits from Cayman Chemicals (Ann Arbor, MI): DNA/RNA Oxidative Damage ELISA Kit (cat. # 589320; detection limit 30 pg/mL), 8-Isoprostane ELISA Kit (cat. # 516351; detection limit 2.7 pg/mL), and Antioxidant Assay Kit [cat. # 709001; detection limit 44 μM (0.044 mM) Trolox equivalents]. (The DNA/RNA Oxidative Damage kit measures all three oxidized guanine species, namely 8-OHdG, 8-hydroxyguanosine, and 8-hydroxyguanine; this cumulative measurement is referred to in this study as 8-OHdG. The Antioxidant Assay Kit measures the cumulative effects of endogenous and food-derived antioxidants.) HSPA8 was measured in single wells using undiluted CSF.

8-hydroxy-2′-deoxyguanosine and 8-ISO were measured in duplicate after diluting samples 1:4 and 1:2, respectively, with EIA buffer. TAC was measured in duplicate in undiluted samples. The concentrations of 8-OHdG, 8-ISO, and TAC used in statistical analyses were mean values of duplicate measurements. Two ELISA plates were used for HSPA8 measurements, four plates were used for 8-OHdG measurements, three plates were used for 8-ISO measurements, and three plates were used for TAC measurements. The coefficients of variation (CV), calculated only for the 8-OHdG, 8-ISO, and TAC assays, were 3.6, 2.0, and 10.4.

### Statistical Analyses

HSPA8, 8-OHdG, 8-ISO, and TAC concentrations were summarized with medians, interquartile ranges, and ranges. The associations between these measures, and between each measure and subject age, were examined with Spearman’s rank-order correlation coefficient (Spearman’s rho). Gender differences for each measure were explored with non-parametric summaries, Wilcoxon’s rank sum test, and scatter plots which included LOWESS (“locally weighted scatterplot smoother”) lines. Because the males in this study tended to be younger than the females, the non-graphical comparisons of data between genders used data only from subjects at least 40 years old (9 men and 16 women, with median ages of 60 and 64, respectively). Statistical significance was set at *p* < 0.05 for all analyses; *p*-values were not adjusted for multiple testing. The SAS System for Windows version 9.3 (SAS Institute Inc., Cary, NC, USA) and Minitab release 14 (Minitab Inc., State College, PA, USA) were used for statistical analysis.

## Results

### Patient Demographics

Our study subjects consisted of 18 males and 16 females. Their mean age (±SD) was 51.3 ± 17.9 years, with a range from 20 to 75 years.

### HSPA8, 8-OHdG, 8-ISO, and TAC Concentrations in CSF

Summary statistics (medians, 25th and 75th percentiles, and ranges) for CSF HSPA8, 8-OHdG, 8-ISO, and TAC concentrations are shown in **Table 1**. All four of these measures were detected in all samples, although some of the HSPA8 and 8-ISO measurements were only slightly greater than the lower limits of sensitivity for their respective assays (lowest value for HSPA8 = 0.14 ng/mL, lower limit of sensitivity = 0.128 ng/mL; lowest value for 8-ISO = 3.2 pg/mL, lower limit of sensitivity = 2.7 pg/mL). Comparison of the two oxidative stress biomarkers indicated that 8-OHdG concentrations (median = 784.9 pg/mL) were far higher than those for 8-ISO (median = 6.5 pg/mL).

**Table 1:** Summary statistics for HSPA8, 8-OHdG, 8-ISO, and total antioxidant capacity (TAC) concentrations in CSF from 34 healthy subjects.

Measure	Units	Median	25th percentile	75th percentile	Range
HSPA8	ng/mL	0.45	0.36	0.64	0.14–0.95
8-OHdG	pg/mL	784.9	712.5	922.7	416.9–1159.1
8-ISO	pg/mL	6.5	5.3	8.7	3.2–16.7
TAC	mM	0.38	0.33	0.40	0.24–0.59

*HSPA8, heat shock 70-kDa protein 8; 8-ISO, 8-isoprostane; 8-OHdG, 8-hydroxy-2′-deoxyguanosine; TAC, total antioxidant capacity.*

### Associations between Subject Age, HSPA8, Oxidative Stress Biomarkers, and Total Antioxidant Capacity in CSF

The associations involving subject age and the other measures are shown in **Table 2**. Age was negatively associated with HSPA8 (ρ = –0.47; *p* = 0.005) and positively associated with 8-OHdG (ρ = 0.61; *p* = 0.0001). Age was weakly associated with 8-ISO (ρ = 0.16) and TAC (ρ = 0.10). HSPA8 was negatively correlated with 8-OHdG (ρ = –0.58; *p* = 0.0004), but poorly correlated with 8-ISO (ρ = –0.15) and TAC (ρ = 0.004). 8-OHdG, 8-ISO, and TAC were poorly correlated with each other (ρ values: 8-OHdG vs. 8-ISO: 0.12; 8-OHdG vs. TAC: 0.17; 8-ISO vs. TAC: –0.29).

**Table 2:** Associations between age, HSPA8, 8-OHdG, 8-ISO, and TAC in CSF from 34 healthy subjects.

	HSPA8	8-ISO	8-OHdG	TAC
Age	–0.469	0.165	0.614	0.100
	0.005	0.35	0.0001	0.57
HSPA8		–0.148	–0.576	0.004
		0.40	0.0004	0.98
8-ISO			0.117	–0.287
			0.51	0.10
8-OHdG				0.173
				0.33

*Top number, Spearman’s rho; bottom number, p-value; HSPA8, heat shock 70-kDa protein 8; 8-ISO, 8-isoprostane; 8-OHdG, 8-hydroxy-2′-deoxyguanosine; TAC, total antioxidant capacity.*

### Gender-Related Differences in HSPA8, 8-OHdG, 8-ISO, and TAC

When statistical analyses were limited to subjects ≥ 40 years of age to allow comparison of similar-aged gender cohorts, HSPA8 concentrations were similar between genders (median values for men and women, respectively: 0.44 and 0.40 ng/mL). The concentrations of 8-OHdG and 8-ISO were also similar between genders (median values: 8-OHdG: men, 842 pg/mL, women, 857 pg/mL; 8-ISO: men, 6.00 pg/mL, women, 6.95 pg/mL). However, measurements of TAC suggested that its levels might be higher in men than in women (median values: men, 0.402 mM, women, 0.366 mM; *p* = 0.018).

**FIGURE 1:**
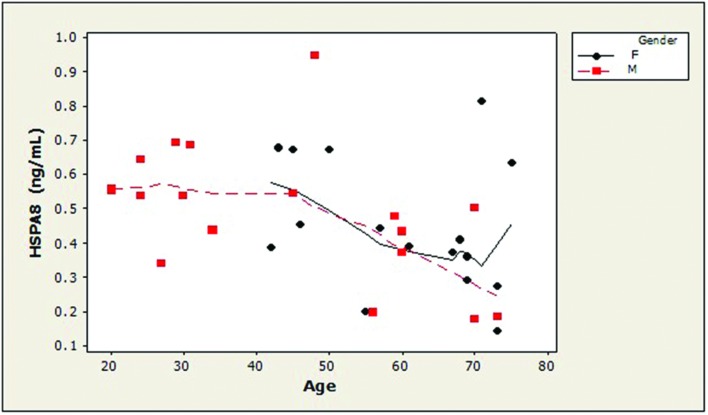
**Distribution of CSF HSPA8 concentrations as a function of age in healthy adults**. HSPA8 concentration was negatively correlated with subject age (Spearman’s ρ = –0.47; *p* = 0.005). LOWESS curves (dashed lines, men; solid lines, women) suggested that HSPA8 may decrease similarly in men and women after age 45. An age-related decrease in HSPA8 was not seen in men prior to age 45. (HSPA8, heat shock 70-kDa protein 8).

**FIGURE 2:**
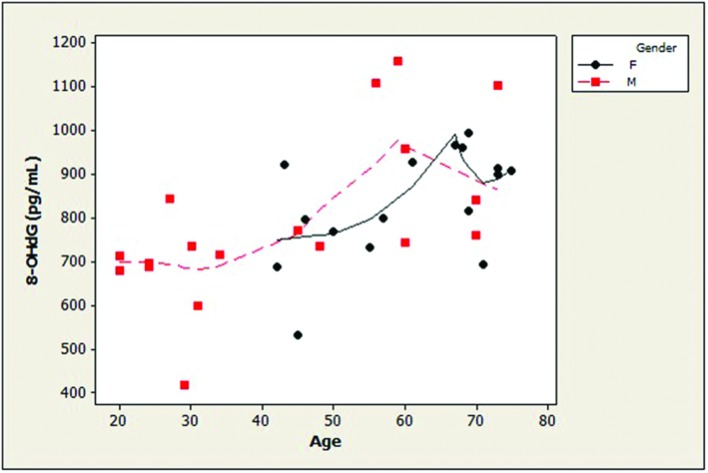
**Distribution of CSF concentrations of 8-OHdG as a function of age in healthy adults**. Age and 8-OHdG were strongly positively associated (ρ = 0.61; *p* = 0.0001). 8-OHdG levels tended to increase with age with similar patterns for both genders. (dashed lines, men; solid lines, women; 8-OHdG, 8-hydroxy-2′-deoxyguanosine).

Examination of the slopes of LOWESS lines revealed that HSPA8 concentrations appeared to decline similarly with age, after age 45, in both genders (**Figure 1**). 8-OHdG levels tended to increase with age with similar patterns for both genders (**Figure 2**). The LOWESS lines in the scatterplot of age versus 8-ISO (not shown) suggested a tendency for 8-ISO to increase with age in men beginning at about age 40, but there did not appear to be a relationship between age and 8-ISO levels for women. TAC levels in women (scatter plots not shown) appeared to remain constant during aging, while there was not enough information to draw conclusions regarding possible age-related changes in TAC in men.

## Discussion

Our main finding in this study was that the CSF concentration of HSPA8 decreased with age (ρ = –0.47; *p* = 0.005). An age-related increase in oxidative stress was suggested by the association between age and 8-OHdG (ρ = 0.61; *p* = 0.0001) although no associations with age were found for the other oxidative stress marker 8-ISO or for TAC. HSPA8 was moderately associated with 8-OHdG (ρ = –0.58; *p* = 0.0004). These findings suggest that the age-related increase in oxidative stress in the CNS might contribute to an age-associated decrease in HSPA8.

We found relatively weak correlations between CSF concentrations of 8-OHdG, 8-ISO, and TAC. Our assays for 8-OHdG and 8-ISO evaluated different oxidative parameters; the DNA/RNA Oxidative Damage kit measures nucleic acid oxidation (8-OHdG, 8-OHG, and 8-hydroxyguanine) while our 8-ISO ELISA measures lipid peroxidation. Both 8-OHdG and 8-ISO are widely used as markers for oxidative stress (see reviews by Montuschi et al., 2007 and Gmitterová et al., 2009) and there is no consensus as to which is more sensitive. TAC has also been measured previously in CSF (Lönnrot et al., 1996; Mandrioli et al., 2006). Although TAC is not a measure of oxidative stress, its decrease indicates a reduction in antioxidant protection, which could result in increased oxidative stress. Among these three markers, we found only 8-OHdG to be moderately associated, in all study subjects, with subject age (ρ = 0.61). [Although 8-ISO was strongly positively correlated with age in men ≥40 (ρ = 0.84) and 8-OHdG and 8-ISO were moderately associated in this subset (ρ = 0.53), these results were based on data from only nine men.] Our results suggest that 8-OHdG may be more sensitive than 8-ISO or TAC for detecting oxidative stress in CSF. Previous studies suggested that measurements of 8-OHdG and 8-ISO can be used to detect age-related increases in oxidative stress in CSF. Guest et al. (2014) reported an increase of 15% in 8-OHdG and 5.7% in F2-isoprostane in CSF between normal subjects ≤ 45 years old and those > 45 years old, and Peskind et al. (2014) found a 10% increase in F2-isoprostanes from age 45 to age 71. Montine et al. (2002, 2011) also found an age-related increase in F2-isoprostanes in one study but not in a different study.

The mitochondrial respiratory chain is the main source of reactive oxygen species (ROS) for autophagy signaling (Osellame and Duchen, 2014), so our finding of a negative association (ρ = –0.58; *p* = 0.0004) between the oxidative stress marker 8-OHdG and HSPA8 may be relevant to the selective removal of mitochondria by autophagy, which is termed mitophagy. This process is initiated by damaged mitochondria (Osellame and Duchen, 2014). Chronic impairment of mitochondrial function results in extensive mitochondrial production of ROS, which shift from non-selective autophagy inducers to the more selective process of mitophagy in an effort to save the cell by removing injured mitochondria (Filomeni et al., 2015). ROS can cause irreversible oxidative modification and loss of function to mitochondrial proteins, lipids, and DNA. Paradoxically, while mild oxidative stress may trigger mitophagy (Frank et al., 2012), increased oxidative stress, paired with apoptotic proteases, can also inactivate mitophagy, allowing cell death mechanisms to proceed (Kubli and Gustafsson, 2012). Whether the negative association we found in CSF between oxidative stress (as indicated by 8-OHdG) and HSPA8 might indicate a similar correlation between oxidative stress and mitophagy is unknown.

Oxidative stress is increased in postmortem brain specimens from healthy elderly individuals (Smith et al., 1991; Mecocci et al., 1993; Agarwal and Sohal, 1994; Dei et al., 2002). The influence of normal aging, and its associated increase in oxidative stress, on autophagy in the human CNS is unknown. Our findings suggest that CSF concentrations of the autophagy-associated molecular chaperone HSPA8 may decrease with age. If these results can be confirmed in a larger cohort, then the relationship of CSF HSPA8 to its brain levels should be examined. If the age-related alteration in CSF HSPA8 reflects a similar change in the brain, then this protein may offer a biomarker for monitoring brain autophagy during normal aging. While HSPA8 alone would likely not be sufficient for this purpose, its measurement could be paired with other autophagy-related proteins such as Beclin 1 and LC3B (Li et al., 2015). Longitudinal monitoring of CSF markers for autophagy might be worthwhile for individuals with known genetic risk factors for neurodegenerative disorders, such as homozygosity for the gene encoding for Apolipoprotein E4, which increases the risk of developing Alzheimer’s disease by 10-fold (Eisenstein, 2011; Rohn et al., 2014) and PD-associated mutations in the *LRRK2* gene.

The interpretation of our results should include the following:

(a) HSPA8 is a multifunctional protein (Liao and Tang, 2014); although a critical protein for CMA, it is not rate-limiting for this process, so its cytosolic concentrations may not correlate with CMA activity (Cuervo and Dice, 2000). It should therefore not be considered as a direct indicator of CMA activity.
(b) Whether our ELISAs detected biologically active HSPA8, or inactive forms of this protein (or its degradation products), is also unknown. Although alterations in the CSF concentrations of HSPA8 might reflect changes in its production in the brain, HSPA8 could also be present in CSF due to release by dying or dead cells.
(c) A limitation of this study is that because our analysis of gender differences for HSPA8 and oxidative measures was performed on data from only 9 men and 16 women in order to compare similar-aged gender cohorts, our findings with regard to possible gender differences for these parameters are preliminary.

We conclude that HSPA8 may decrease in CSF during normal aging, and increased oxidative stress may contribute to this alteration. Further studies, including investigation of the relationship between CSF and brain levels of HSPA8, are indicated to determine the significance of these findings.

## Author Contributions

DL directed the study and prepared the manuscript. AK performed ELISAs, collated the data, and reviewed the manuscript. MC performed the statistical analysis and assisted with manuscript preparation. JA collected the CSF samples and reviewed the manuscript. PL suggested studying HSPA8, and assisted with manuscript preparation.

## Conflict of Interest Statement

The authors declare that the research was conducted in the absence of any commercial or financial relationships that could be construed as a potential conflict of interest.
